# L’enclouage centromedullaire dans les fractures bifocales de la jambe: à propos de 16 cas

**DOI:** 10.11604/pamj.2017.28.139.3036

**Published:** 2017-10-13

**Authors:** Said Zizah, Richard Dolo, Ossama Elassil, Kamal Lahrach, Amine Marzouki, Fawzi Boutayeb

**Affiliations:** 1 Service d’Orthopédie et Traumatologie A, CHU Hassan II, Fès, Maroc

**Keywords:** Fracture bifocale de jambe, enclouage centro-médullaire verrouillé, alésage, Bifocal leg fracture, locked intramedullary nailing, reaming

## Abstract

Afin d'évaluer la gravité de cette lésion inhabituelle, nous avons mené une étude rétrospective intéressant les fractures bifocales fermées du tibia et nous avons étudié la place de l'enclouage centromédullaire ainsi que les différents moyens thérapeutiques. Seize patients avec fractures bifocales de jambe type 42C2 de l'AO ont été traités dans notre unité de traumatologie Orthopédie A du CHU Hassan II Fès. Il s'agissait de sujets jeunes, victimes de traumatisme de moyen à haute énergie. Cinq patients étaient polytraumatisés et deux poly fracturés. L'enclouage centromédullaire verrouillé avec alésage était utilisé dans six cas et sans alésage dans dix cas. Nous avons déplorés après enclouage deux cas de syndromes de loge. Le délai de consolidation moyen était de douze mois. Deux cas de pseudarthroses ont été repris avec succès par un enclouage avec sur alésage. La fracture bifocale de jambe pose de nombreux défis au chirurgien en raison de l'approvisionnement vasculaire précaire du segment intermédiaire et de la grave détérioration des tissus mous environnants. Elles doivent être individualisées de l'ensemble des fractures de jambe tant par leur contexte de survenue que par les difficultés de fixation et la lenteur de leur consolidation.

## Abstract (en)

We conducted a retrospective study of bifocal closed fractures of the tibia in order to assess the severity of this unusual lesion. We analyzed the entry point for intramedullary nailing as well as the different therapeutic options. Sixteen patients with AO type 42C2 bifocal leg fractures were treated in our Traumatology-Orthopedics Unit A at the University Hospital Hassan II, Fez. The subjects were young, victims of medium to high energy trauma. Five subjects were polytraumatized patients and two were poly-traumatized patients with fractures. Six patients underwent locked intramedullary nailing with reaming while seven patients underwent intramedullary nailing without reaming. Two cases of compartment syndrome were recorded after nailing. Mean fracture consolidation time was twelve months. Two cases of pseudarthroses were successfully treated by nailing with reaming. Bifocal leg fractures pose many challenges for the surgeon due to weak vascular supply of the intermediate segment and to severe deterioration of the surrounding soft tissues. They should be detected among the leg fractures based on their context of occurrence, difficulties in fixation as well as slow consolidation time.

## Introduction

La présence de deux foyers fracturaires distincts isolant un segment cortical complet de plusieurs centimètres définit une fracture bifocale de jambe (FBJ). La fréquence des FBJ se situe entre 4 et 6% de l'ensemble des fractures de jambe [1]. Elle fait suite habituellement à un traumatisme de haute énergie associée souvent à des lésions des parties molles [1]. Le traitement des FBJ est exigeant et la technique utilisée pour la stabilisation de la fracture initiale reste controversée. Afin d'évaluer la gravité de cette lésion inhabituelle, nous avons mené une étude rétrospective intéressant les fractures bifocales fermées du tibia et nous avons étudié la place de l'ECM ainsi que les différents moyens thérapeutiques.

## Méthodes

**Figure 1:**
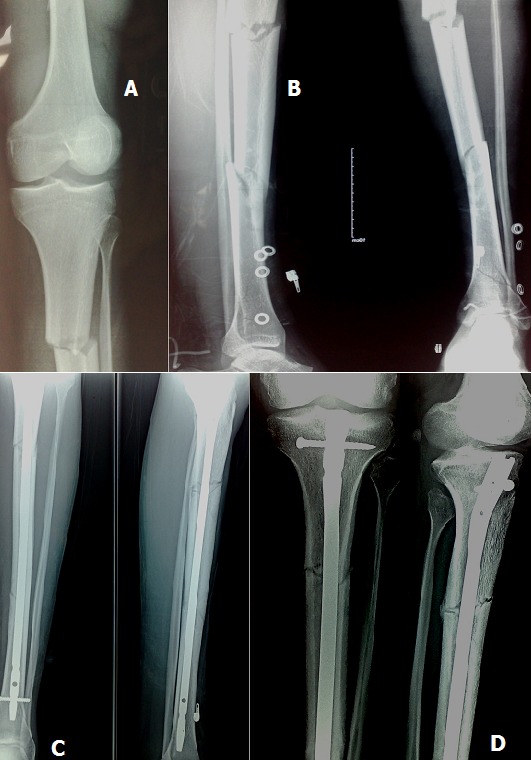
(A, B) fracture bifocale de la jambe type 42C; (C) évaluation à une semaine postopératoire; (D) évaluation à 4 mois postopératoire

De Janvier 2003 à Mai 2012,659 fractures de jambe ont été prises en charge en première intention. Nous avons inclus dans notre étude toutes les fractures bifocales de jambe fermées ou ouvertes stade I Cauchoix Duparc sans lésions vasculaires ou nerveuses, de type 42C2 selon la classification de l'AO [2] soit toute fracture comportant deux foyers diaphysaires distincts isolant un fragment cylindrique cortical de plusieurs centimètres de long. Nous avons trouvé 16 FBJ répondant à nos critères d'inclusion. Un patient a été perdu de vue après le premier contrôle postopératoire. Il y avait 11 hommes et 5 femmes. La moyenne d'âge des patients était de 37 ans (extrêmes 22-67 ans). Les étiologies étaient dominées par les accidents de la voie publique dans 13 cas suivi par 3 cas de chutes de hauteur. Dix fractures ont été fermées et 6 ont été ouvertes. Le côté droit a été atteint dans 75% des cas. Cinq patients étaient polytraumatisés et 2 poly fracturés dont un cas de genou flottant (Figure 1). Seuls 9 cas étaient monotraumatisés ne présentant qu'une FBJ. Selon la classification de l'AO [2], 6 lésions étaient de type C21, 8 de type C22 et 2 de type C23. Les traits de fracture étaient métaphyso-métaphysaire disto-proximal 3 fois, diaphyso métaphysaires proximaux 4 fois, diaphyso-diaphysaires 8 fois et diaphyso métaphysaires distaux 1 fois. La fibula était fracturée dans tous les cas. Le foyer proximal tibial était simple 11 fois, à coin ou comminutif 5 fois et le foyer distal était simple 9 fois, à coin ou comminutif 7 fois. Le foyer proximal n'était pas ou peu déplacé sur les deux incidences 12 fois soit 75%. Le foyer distal était peu ou pas déplacé 6 fois soit 37.5%. La longueur moyenne du segment diaphysaire intermédiaire évaluée sur le cliché de face était de 11.5cm (extrêmes 12cm et 15cm). Le délai opératoire moyen était de 32 heures avec des extrêmes de 6 heures et 72 heures. Deux méthodes opératoires ont été appliquées en urgence: l'enclouage centromédullaire verrouillé statique dans 13 cas et dynamique dans 3 cas. L'enclouage a été réalisé jambe pendante sur table ordinaire avec une traction manuelle et une réduction à foyer fermé sous contrôle scopique. Le diamètre des clous utilisés variaient de 9 à 11 mm de diamètre (10 sans alésage, 6 avec alésage modéré). Une ostéosynthèse fibulaire a été réalisée dans 6 cas (plaque vissée tiers de tube dans 4 cas et embrochage dans 2 cas).

## Résultats

Le délai moyen de consolidation pour la fracture proximale était de 38.8 et de 41.4 semaines pour le foyer distal, il n'y avait aucune différence significative dans le temps de consolidation entre les fractures distales et les fractures proximales. Un cas d'embolie graisseuse a été constaté au deuxième jour avec détresse respiratoire et coma résolutifs. Chez deux patients, une phlébite surale est survenue sans migration embolique pulmonaire. Sur des arguments cliniques, deux patients ont présenté en postopératoire un syndrome de loge imposant des aponévrotomies. Le devenir radiologique est connu pour 15 malades. Les 12 patients traités par un enclouage sans alésage, avaient une réduction complète des deux foyers de fracture. L'appui partiel a été permis en moyenne au troisième mois et totalisé au quatrième. La consolidation était obtenue dans 10 cas en première intention en 5 à 10 mois; trois avaient été dynamisé. Deux patients ont présenté une pseudarthrose (1 du foyer proximal et 1 distal) malgré la dynamisation: ils ont été repris par un enclouage avec suralésage. Quant au résultat clinique, il est connu avec un recul minimum de 18 mois pour 15 patients. La mobilité de la cheville a été jugée strictement comparable au côté opposé dans dix cas. Seuls deux patients ont eu un déficit de mobilité du genou: ils étaient porteurs de lésions épiphysaires fémorales concomitantes.

## Discussion

Peu de travaux ont été consacrés aux FBJ en dehors des publications « historiques » de Boutin [3] et de Decoulx et al [4]. Seuls Woll et Du Wellius [5], Melis et al [6] et Wu et Shih [7] qui ont spécifiquement étudié ces lésions. La FBJ est considéré par de nombreux auteurs une entité lésionnelle particulière [3, 4]. La précarité vasculaire du segment cortical interme'diaire est une des justifications de l'individualisation des FBJ expose'es aux retards de consolidation ou aux pseudarthroses. Elle est souvent associee a une severite accrue des lesions des parties molles. Dans cet te e'tude 37.5% des fractures e'taient ouvertes. Ceci est en accord avec l'etude realisee par Wolet Du welius [5] qui ont rapporte une incidence jusqua 75% des fractures ouvertes dans ce type de lesions. Cette serie confirme les notions epidemiologiques classiques des FBJ: 5% de fractures de jambe traitees dans notre service etaient bifocales soit une moyenne de 3 par an. 40% des patients etaient polytraumatises ou polyfractures. La notion de traumatisme de haute energie sillustre par la frequence des lesions ouvertes (37.5%) et des syndromes de loge (12.5%). Les diverses methodes therapeutiques proposees pour les FBJ sont analysees avec plus ou moins de precision dans la litterature. La fixation externe est apparue techniquement difficile tant pour reduire que stabiliser deux foyers de fracture et ce malgre les poignees orientables ou ladjonction de fiches complementaires. La lenteur de la consolidation osseuse des FBJ fait courir un risque septique local sur les fiches avec risque de perte de rigidite du montage. De meme, il est impossible dobtenir une dynamisation preferentielle de l'un ou l'autre foyer. Par contre, le fixateur externe est aisement convertissable en enclouage apres une periode dattente de cicatrisation et de sterilisation des orifices des fiches [8, 9]. L'enclouage sans alesage a demontre une bonne efficacite réductionnelle mais son incapacite a consolider les deux foyers avec plus d'une pseudarthrose sur deux. Il est facilement repris par un enclouage avec alesage. Sa meilleure indication est une fracture fermée ou moderement ouverte compliquee d'un syndrome de loge immediat qui impose des aponevrotomies en meme temps que l'enclouage. L'enclouage avec alesage modéré nous semble devoir être privilegié dans la plupart des cas avec un montage statique.

La surveillance radiologique mensuelle doit etre stricte pour programmer l'appui, mais aussi la dynamisation par ablation des vis voire le changement de clou. Lors du changement de clou avec suralesage, losteotomie fibulaire et le type de montage se discutent au cas par cas, car on se trouve souvent devant un seul foyer non consolide [10]. Le type anatomique meme de la FBJ influe sur le mode d'ostéosynthèse en particulier la localisation métaphysaire d'un des foyers. Melis et al [6] et Muleretal [2]. ont bien percu la necessite dintegrer dans la classification de ces fractures la situation des deux foyers. Les fractures proximales sont particulierement dificiles a reduire et les distales a stabiliser. Pour les premieres, il s'agit d'une limite d'indication de l'enclouage [11]. Pour les metaphysaires distales, il est necessaire dutiliser des clous specifiques dont les orifices de verrouillage sont situe's plus distalement et/ou fixer la fibula si elle est porteuse d'un trait au meme niveau [12]. Les taux declare's d'infection des FBJ ouvertes vont de 21% pour l'ECM sans alesage a 8% lorsqu'il est alese [1]. La nature segmentaire de la fracture peut augmenter le risque d'infection. Dans notre serie, le taux d'infection est reste dans un pourcentage acceptable comparable a celui rapporte par Jennyetal [13] et Court-Brownetal [14]. Le syndrome des loges a ete signale dans pres de 50% des cas de FBJ [5], mais d'autres auteurs n'ont pas trouve de telles complications [15]. Dans notre serie, 2 patients ont presente en post-operatoire un syndrome de loge, ce qu'il a situe dans les taux habituels [16, 17]. Plusieurs facteurs ont été évoqués: effort réductionnel en traction, compression du creux poplité, alésage. La contusion musculaire directe, et l'hématome périfracturaire augmentent la pression dans les loges: ces deux arguments sont avancés par WuetShih [7] pour recommander l'enclouage des FBJ apre's quelques jours de traction. Lors d'un enclouage avec alesage l'enregistrement systematique des pressions endomedullaires permet de constater l'augmentation des pressions dans les loges a chaque passage et de poser l'indication d'aponevrotomie [18, 19]. L'enclouage sans alesage entraine une hyperpression unique lors de la reduction et de l'enfoncement du clou [20]. Dans nos 2cas de syndrome de loge diagnostiques immediatement, des aponevrotomies ont ete realisees et les fractures ont ete enclouees avec passage de la seule premiere tete d'alesage comme le recommandent Torneta et Templeman [21]. Le caractere comminutif du foyer, sa situation distale, des foyers tres deplaces et un segment intermediaire cortical court semblent etre des facteurs de pseudarthrose. Les delais de consolidation des FBJ lorsquon considere les deux foyers sont denviron le double que pour une fracture type A ou B. Cette etude a montre que la consolidation se faisait de maniere dissociee, un des foyers consolidait dans des delais habituels et l'autre stagnait. L'alesage a ete rendu responsable d'augmenter la devascularisation du foyer et dans le cas particulier d'une FBJ du fragment intermédiaire [22]. Des travaux experimentaux ont montre que le retablissement du flux vasculaire endocortical se faisait rapidement apres alesage [23, 24]. Par aileurs l'alesat aurait un role osteogenique [24].

## Conclusion

La FBJ pose de nombreux defis au chirurgien en raison de l'approvisionnement vasculaire precaire du segment intermediaire et la grave deterioration des tissus mous environnants. Les FBJ doivent etre individualisees de l'ensemble des fractures de jambe tant par leur contexte de survenue que par les dificultes de fixation et la lenteur de leur consolidation.

### Etat des connaissances actuelles sur le sujet

- La fracture bifocale de la jambe est une entité lésionnelle particulière;
- Taux élevé des complications immédiates et secondaires;
- Le traitement des FBJ est exigeant et la technique utilisée pour la stabilisation de la fracture initiale reste controversée.

### Contribution de notre étude à la connaissance

- Evaluation de la gravité de cette lésion inhabituelle;
- Les FBJ doivent etre individualise'es de l'ensemble des fractures de jambe tant par leur contexte de survenue que par les difficulte's de fixation et la lenteur de leur consolidation.

## Conflits d’intérêts

Les auteurs ne déclarent aucun conflit d'intérêts.
